# Supplementary material for: SUMO targets the APC/C to regulate transition from metaphase to anaphase
Source: Nat Commun. 2018 Mar 16;9:1119. doi: 10.1038/s41467-018-03486-4 (PMC5856775; doi:10.1038/s41467-018-03486-4)
Supplement: Supplementary file 1 — Description of Additional Supplementary Files(PDF 174 kb) [file 41467_2018_3486_MOESM1_ESM.pdf]

Description of Additional Supplementary Files

File Name: Supplementary Data 1
Description: Mass spectrometric analysis of the APC/C complex purified via GFPTrap. The APC/C complex was purified via GFP-Trap from HeLa cells expressing GFP-tagged APC4 WT or K772,798R mutant. Subunits of the APC/C complex are marked in yellow.

File Name: Supplementary Data 2
Description: Mass spectrometric analysis of the purification of the endogenous APC/C complex. The endogenous complex was purified via immunoprecipitation with anti-CDC27 antibody from HeLa cells. Afterwards, the purified proteins were either SUMOylated in vitro (+SUMO) or treated with the SUMOylation machinery lacking UBC9 as a control (-SUMO). Subunits of the APC/C complex are marked in yellow. The values for SUMO are shown in red.

File Name: Supplementary Data 3
Description: Mass spectrometric analysis of proteins binding to non-SUMOylated and SUMOylated APC/C. Proteins were purified via pulldown of recombinant APC/C harboring either Strep-tagged APC4 WT or Strep-tagged APC4 K772,798R mutant. Values for KIF18B and RacGAP1 are shown in green. Values for subunits of the APC/C complex are shown in yellow.
