# Supplementary material for: SUMO targets the APC/C to regulate transition from metaphase to anaphase
Source: Nat Commun. 2018 Mar 16;9:1119. doi: 10.1038/s41467-018-03486-4 (PMC5856775; doi:10.1038/s41467-018-03486-4)
Supplement: Supplementary file 2 — Supplementary Information(PDF 6682 kb) [file 41467_2018_3486_MOESM2_ESM.pdf]

SUMO targets the APC/C to regulate transition from metaphase to anaphase

Eifler, K. et al.

Supplementary Information

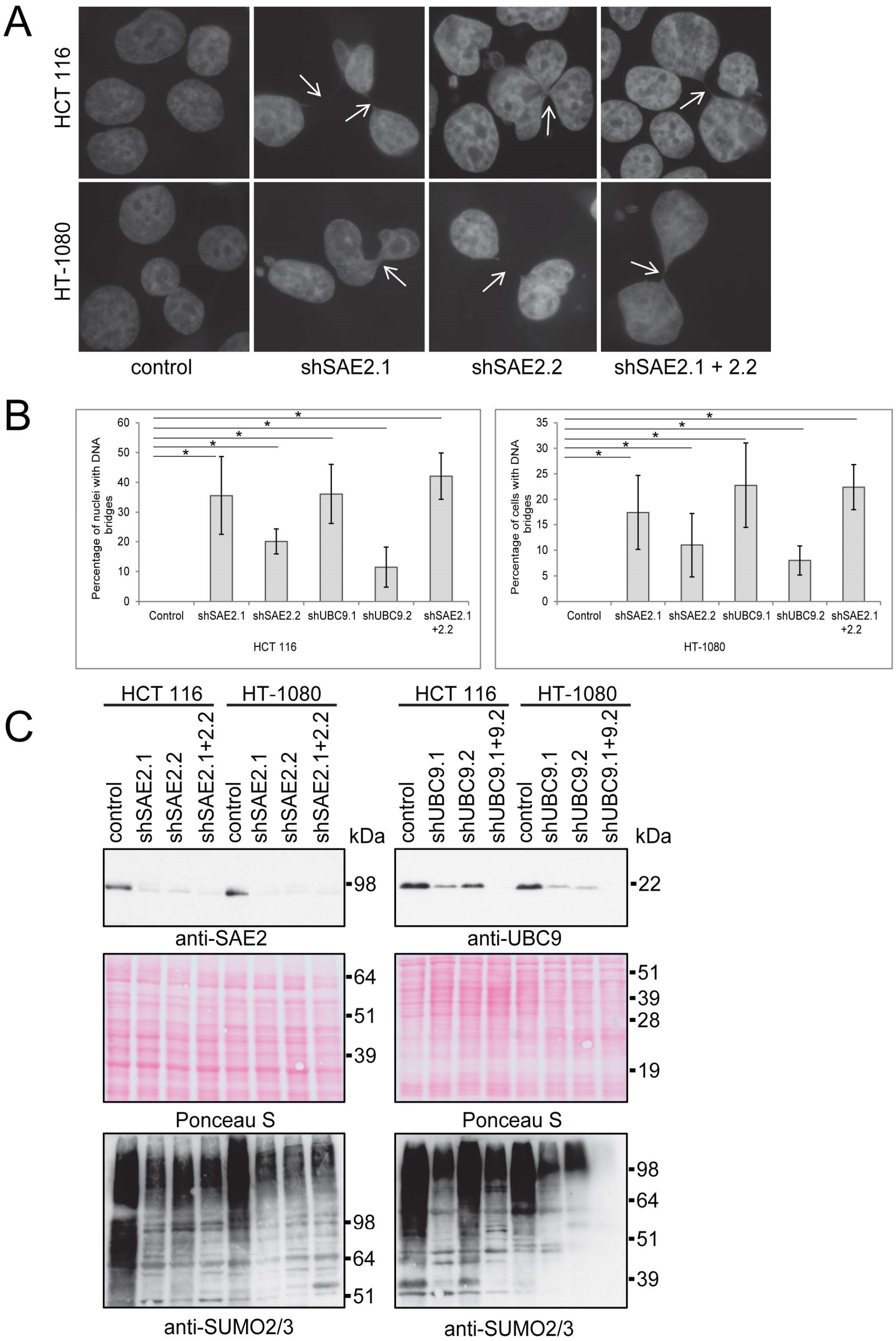

Supplementary Fig. 1

Supplementary Fig. 1: Knockdown of the SUMOylation pathway induces the formation of chromatin bridges in HCT116 and HT-1080 cells

A) HCT116 and HT-1080 cells were treated with lentivirus expressing shRNAs against subunit 2 of the SUMO Activating Enzyme for 72 hours. Cells were fixed and stained with Hoechst to visualize the DNA and the formation of DNA bridges (white arrows). Scale bars correspond to 10 µM. B) Cells were treated with lentivirus expressing shRNAs against the SUMO activating enzyme (SAE) or the SUMO conjugating enzyme (UBC9). The percentage of cells with DNA bridges visualized via microscopy and standard deviations were calculated for 100 cells per condition resulting from four independent experiments. A two-sided Student's *t*-test was performed. p-values: *<0.005. C) The amount of SAE2, UBC9 and SUMO2/3 conjugates was monitored by immunoblot analysis of total cell lysates, 72 h after infection with the lentivirus. Equal loading was confirmed via Ponceau S staining.

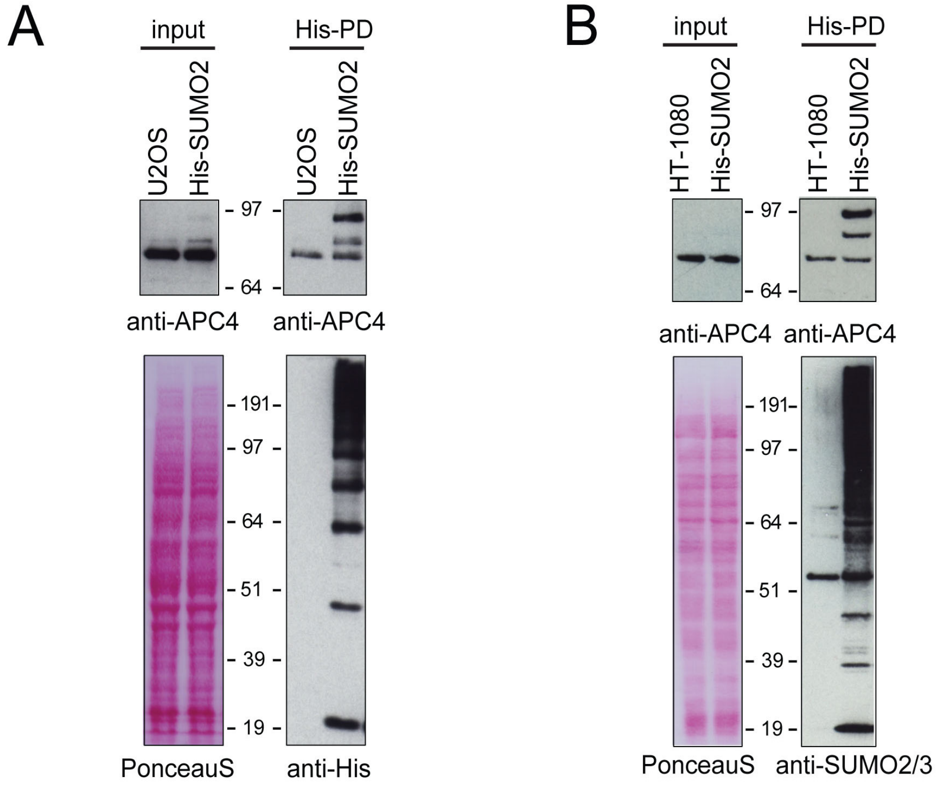

Supplementary Fig. 2

Supplementary Fig. 2: Posttranslational modification of human APC4 by SUMOylation

A) U2OS cells and U2OS cells stably expressing His-tagged SUMO2 (His-SUMO2) were subjected to His-pulldown experiments (His-PD). Total lysates and pulldown samples were analyzed via immunoblotting to monitor APC4 and SUMO2/3 conjugates. Equal loading was confirmed via Ponceau S staining. B) HT-1080 cells and HT-1080 cells infected with a lentivirus expressing His-SUMO2 were lysed for His-pulldown 72 h after infection. Total lysates and pulldown samples were analyzed via immunoblotting as described above. Experiments were performed twice and representative results are shown.

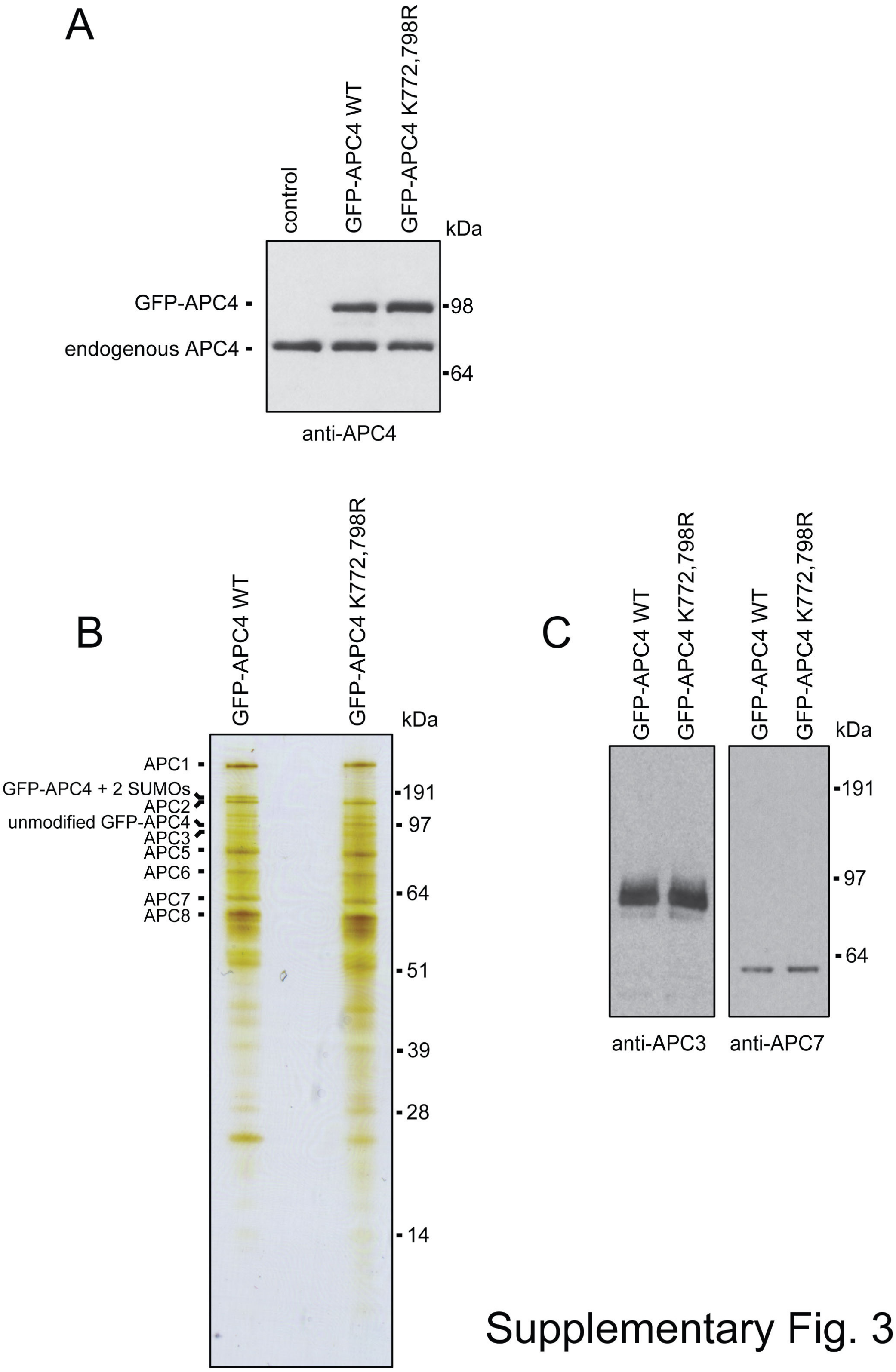

Supplementary Fig. 3

Supplementary Fig. 3: K772 and K798 of APC4 are the main SUMOylation sites within the APC/C complex

A) HeLa cells were infected with control virus or retrovirus expressing either GFP-APC4-WT or GFP-APC4-K772,798R. Cells were selected for puromycin resistance and lysates were analyzed via immunoblotting seven days after infection. B) The APC/C complex was purified from cells infected with retrovirus expressing either GFP-APC4-WT or GFP-APC4-K772,798R via GFP-trap and SUMOylated *in vitro*. Samples were eluted from the beads, loaded on an SDS-PAGE and silverstained. C) APC/C complex purified from cells infected with retrovirus expressing either GFP-APC4-WT or GFP-APC4-K772,798R via GFP-trap and SUMOylated *in vitro,* was analyzed by immunoblotting with anti-APC3 and anti-APC7 antibody. This figure shows representative results from two individual experiments.

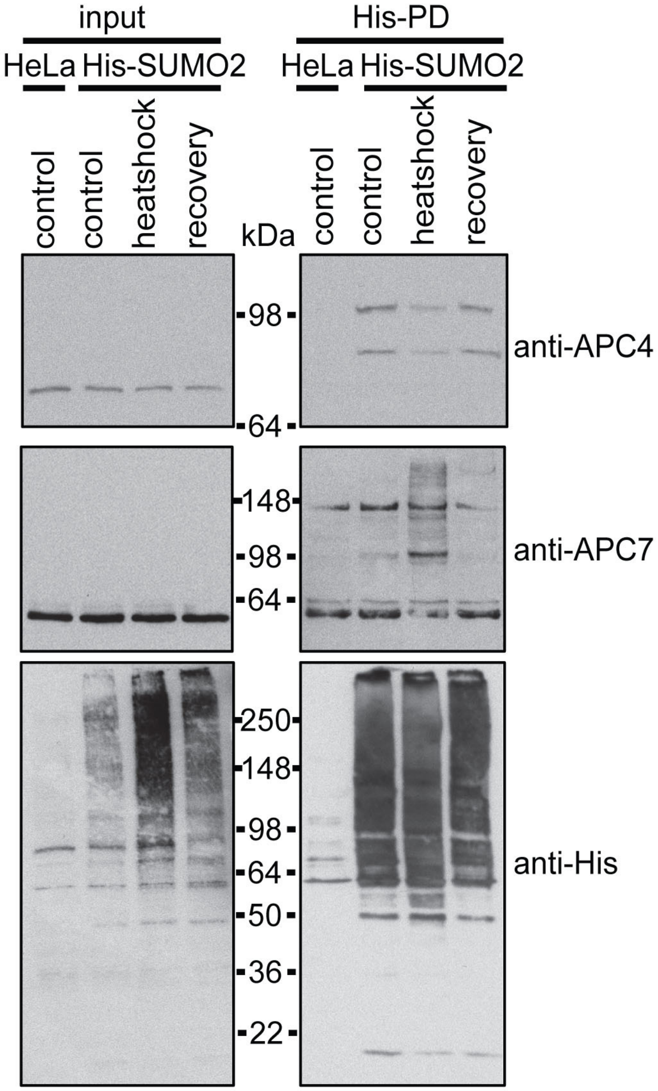

Supplementary Fig. 4

Supplementary Fig. 4: APC7 is only SUMOylated after heatshock

HeLa cells expressing His-tagged SUMO2 (His-SUMO2) were either not treated, subjected to heat shock for 75 min at 43°C and lysed directly (heat shock) or lysed 2 hours after heat shock (recovery). SUMO2 conjugates were purified via His-pulldown and SUMOylation of the APC/C subunits APC4 and APC7 was visualized by immunoblotting.

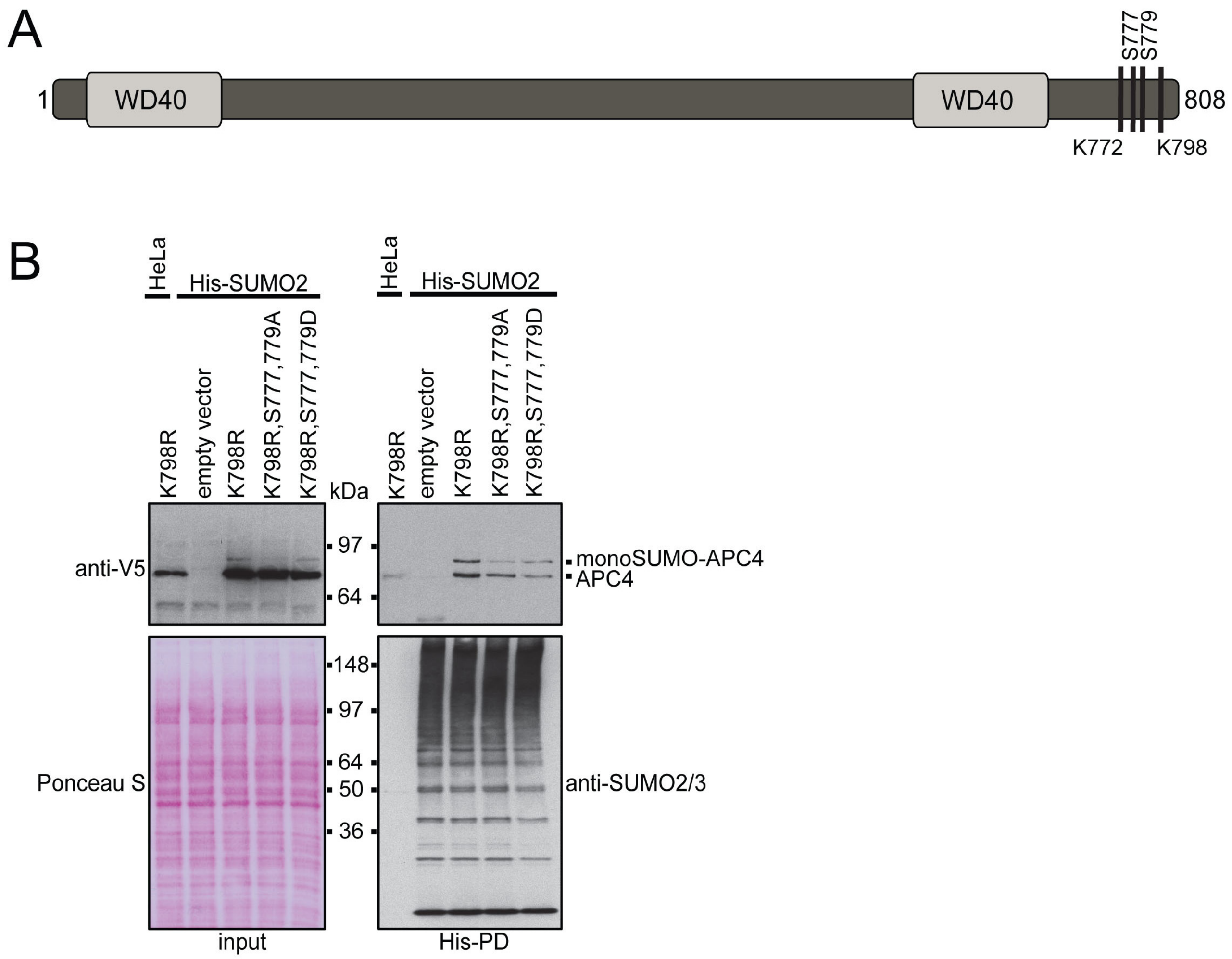

Supplementary Fig. 5

Supplementary Fig. 5: K772 of APC4 is located in a phosphorylation dependent SUMOylation motif

A) Schematic overview on APC4 highlighting the SUMOylated lysine residues and the adjacent phosphorylated serine residues.

B) HeLa cells expressing His-tagged SUMO2 (His-SUMO2) were transfected with mutants of APC4 harboring an N-terminal V5-tag. SUMO2 conjugates were isolated via His-pulldown and input and pulldown samples were analyzed via immunoblotting with anti-V5 and anti-SUMO2/3 antibodies.

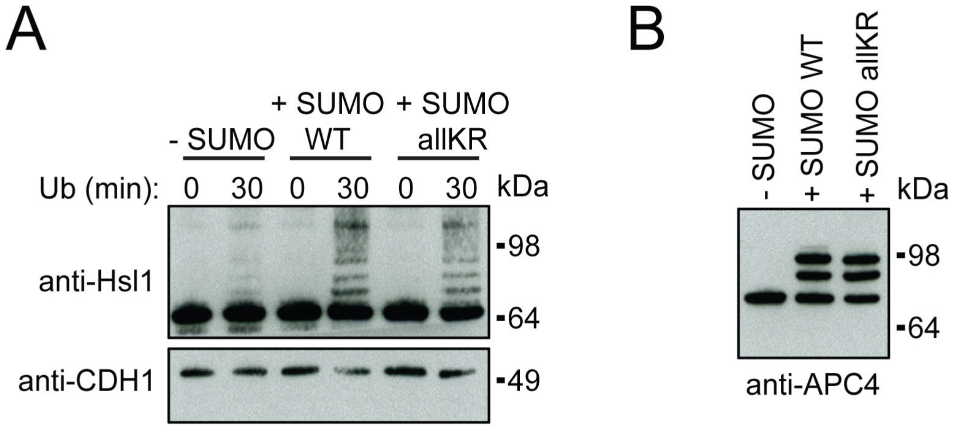

Supplementary Fig. 6

Supplementary Fig. 6: SUMOylation enhances ligase activity towards Hsl1

A) Endogenous APC/C complex was purified from HeLa cells via immunoprecipitation and either mock-treated without UBC9, SUMOylated with wildtype SUMO or SUMOylated with a mutant SUMO where all lysines were mutated to arginines. Ubiquitylation of Hsl1 was monitored via immunoblotting with anti-Hsl1 antibody. Equal levels of CDH1 were confirmed by immunoblotting with anti-CDH1 antibody. B) *In vitro* SUMOylation of APC4 was confirmed via immunoblotting with anti-APC4 antibody.

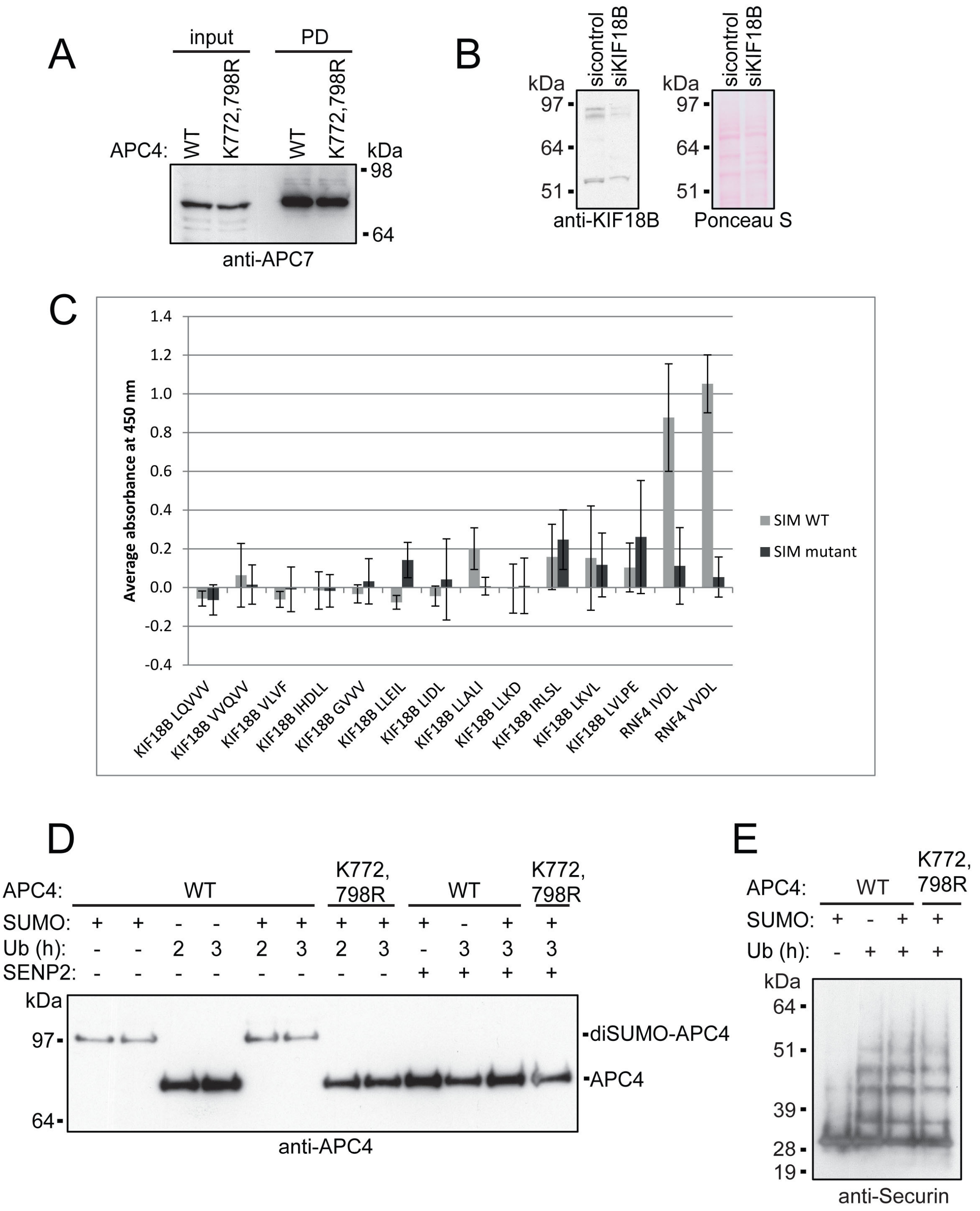

Supplementary Fig. 7

Supplementary Fig. 7: Ubiquitylation of KIF18B is enhanced after SUMOylation of APC4

A) Immunoblot analysis with anti-APC7 antibody of HeLa lysate and samples taken after co-purification of potential binders with the Strep-tagged APC/C wildtype and mutant shows equal amounts of this APC/C subunit. B) HeLa cells were transfected with a control siRNA (sicontrol) or an siRNA against KIF18B (siKIF18B) and cell lysates were analyzed via immunoblot with anti-KIF18B antibody 72 h after transfection. Equal amounts of protein were confirmed via Ponceau S staining C) Twelve different KIF18B peptides and their corresponding putative SIM mutant peptides were fused to biotin and a PEG linker and immobilized on avidin coated wells. The peptides were incubated with recombinant 3xSUMO2 chains and an anti-SUMO2/3 ELISA was performed to determine the binding ability of these peptides to 3xSUMO2 chains. As a control, SIM-containing peptides of RNF4 and their respective SIM mutant peptides were tested. Mean values and standard deviations were calculated from three independent experiments. D) Recombinant APC/C complex harboring either APC4 WT or the APC4 K772,798R double mutant was SUMOylated *in vitro.* Subsequently, *in vitro* ubiquitylation activity was studied using KIF18B as substrate. To monitor the SUMOylation levels of APC4, samples were analyzed via immunoblotting with an anti-APC4 antibody. E) Similar *in vitro* ubiquitylation assays were performed with recombinant APC/C complex harboring either APC4 WT or the APC4 K772,798R double mutant and recombinant Securin as substrate. Ubiquitylation of Securin was monitored via immunoblot analysis making use of anti-Securin antibody.

Supplementary Fig. 8: Full scans of blots

Figure 1C

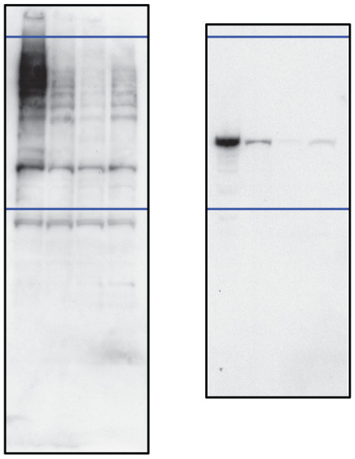

anti-SUMO2/3 anti-SAE2

Figure 2B

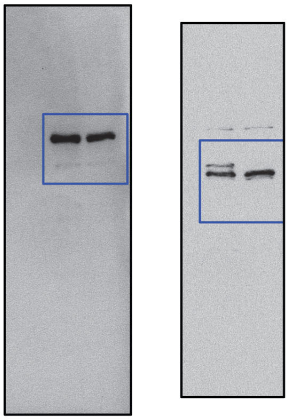

anti-APC2 anti-CUL4A

Figure 2C

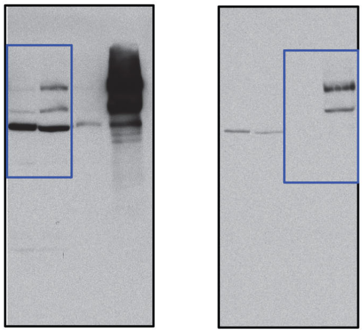

anti-APC4 long exposure

anti-APC4 short exposure

Figure 3B

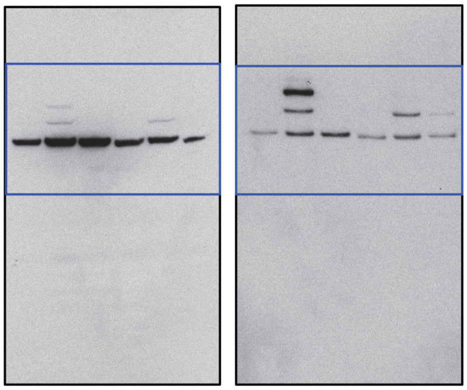

anti-V5 input

anti-V5 His-PD

Figure 4B

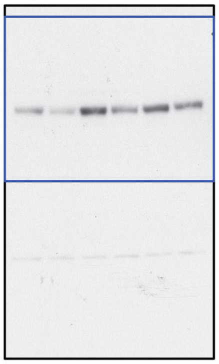

anti-APC4

Figure 5C

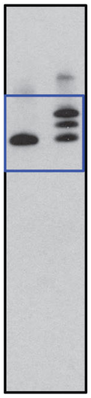

anti-APC4

Figure 5D

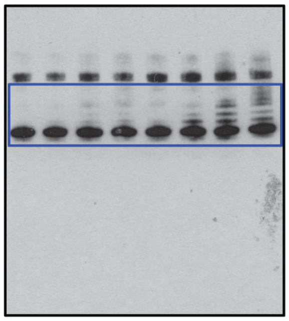

anti-Hsl1

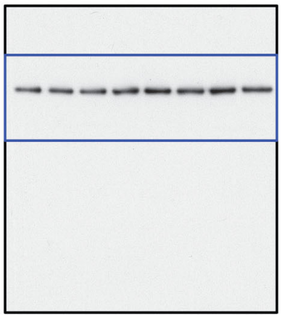

anti-APC2

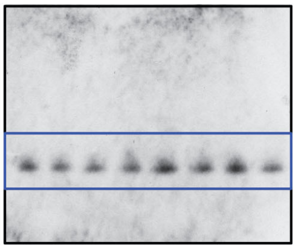

anti-APC11

Figure 6B

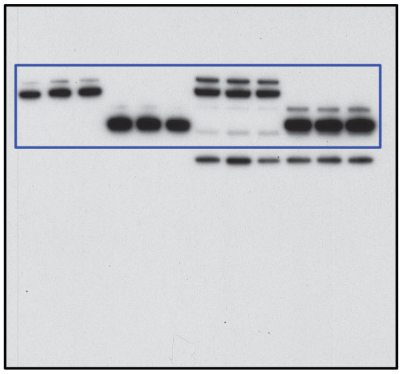

anti-APC4

Figure 7A

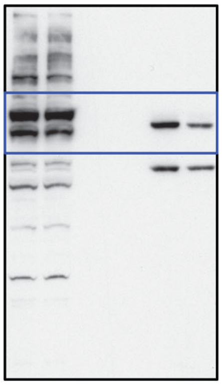

anti-KIF18B

Figure 7B

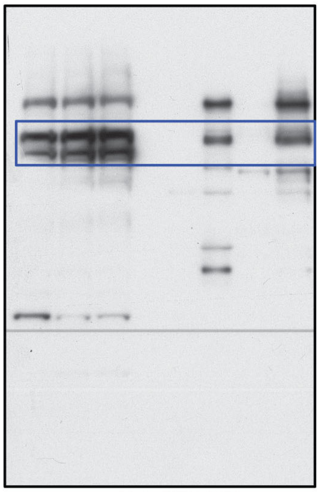

anti-KIF18B

Figure 7D

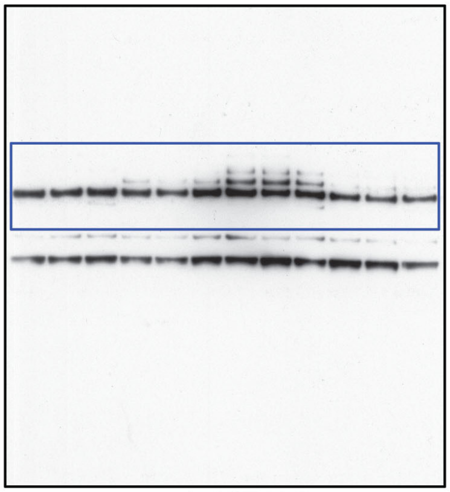

anti-KIF18B

Supplementary Fig. 1C

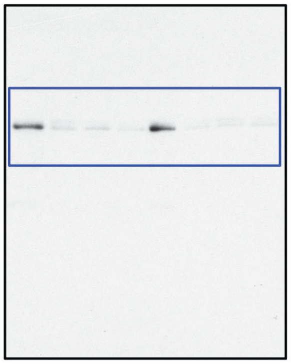
anti-SAE2

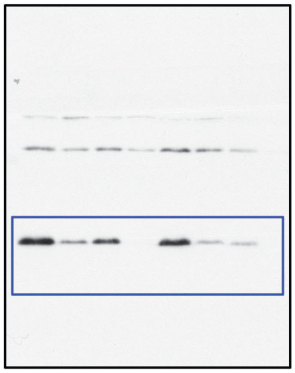
anti-UBC9

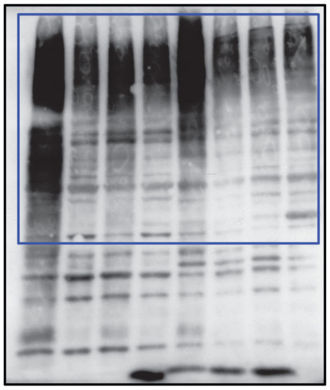
anti-SUMO2/3

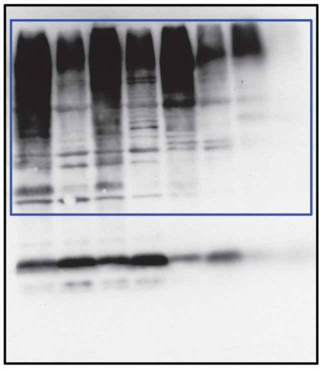
anti-SUMO2/3

Supplementary Fig. 2A

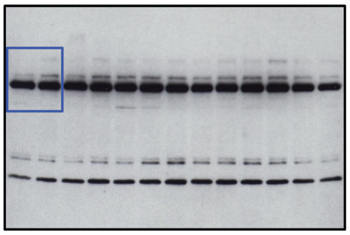
anti-APC4
input

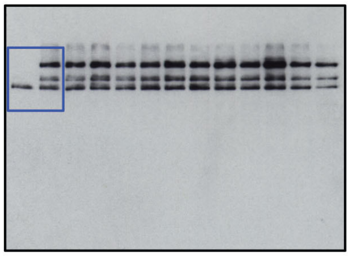
anti-APC4
His-PD

Supplementary Fig. 2B

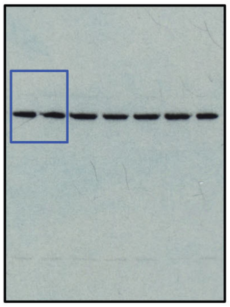
anti-APC4
input

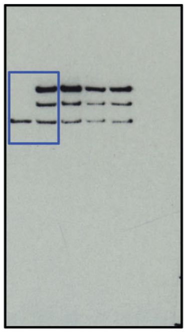
anti-APC4
His-PD

Supplementary Fig. 3A

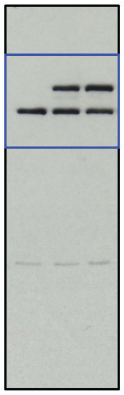

anti-APC4

Supplementary Fig. 3C

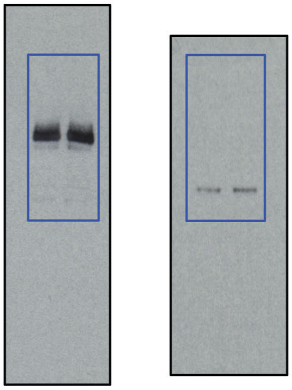

anti-APC3 anti-APC7

Supplementary Fig. 4

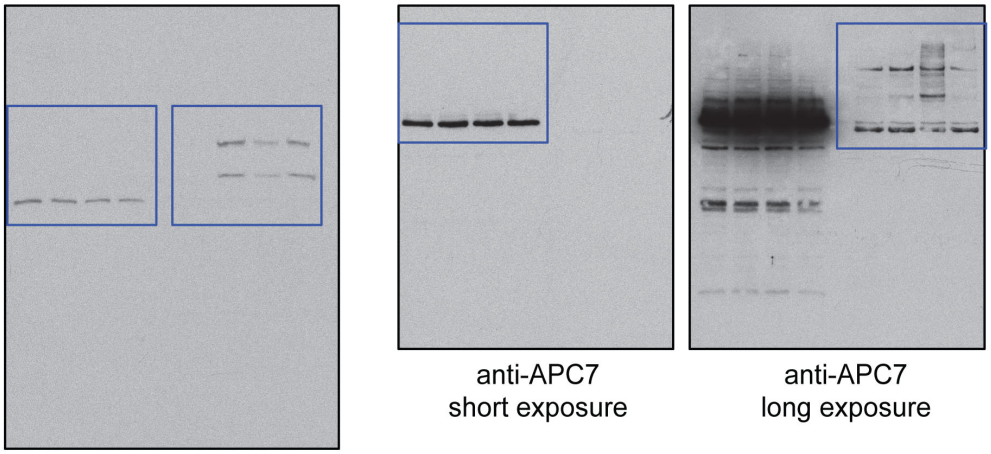

anti-APC4

anti-APC7
short exposure

anti-APC7
long exposure

Supplementary Fig. 5

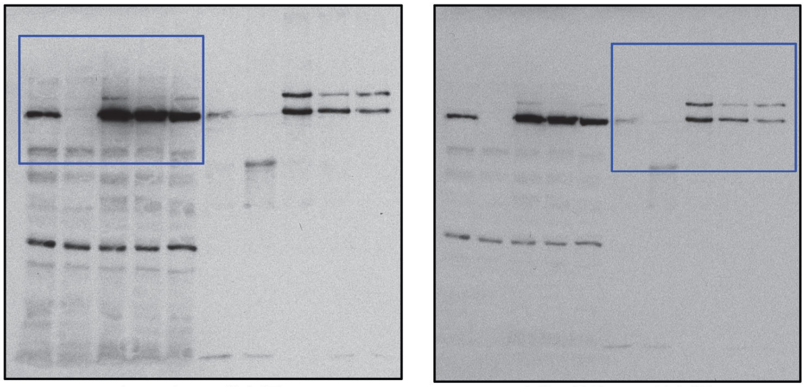

input, anti-V5
long exposure

His-PD, anti-V5
short exposure

Supplementary Fig. 6

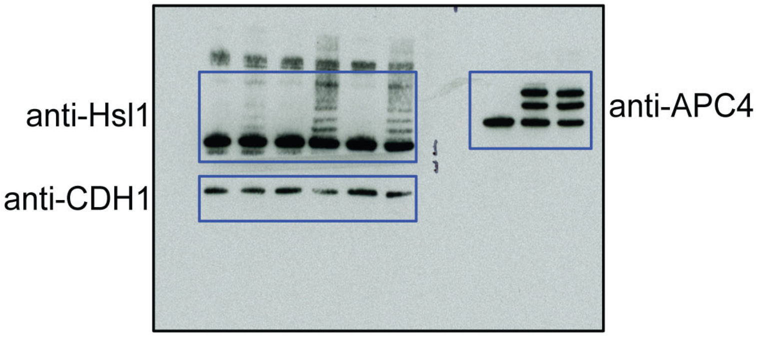

Supplementary Fig. 7A

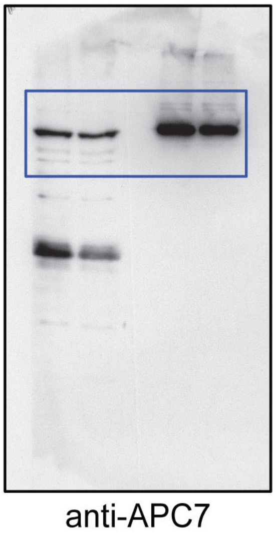

Supplementary Fig. 7B

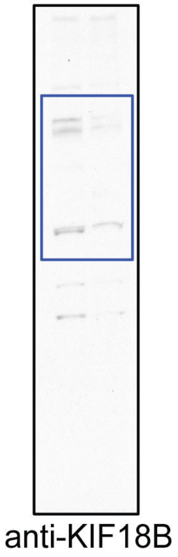

Supplementary Fig. 7D

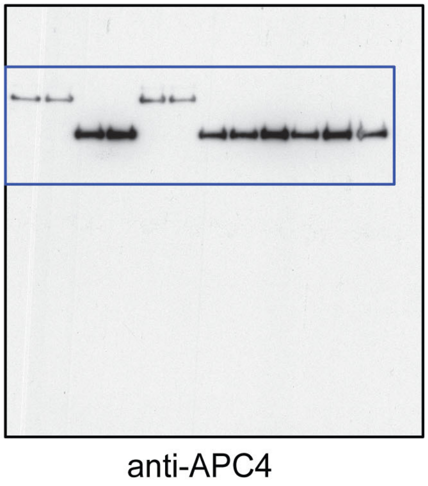

Supplementary Fig. 7E

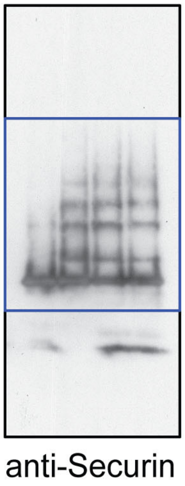

Supplementary table 1: List of putative SUMO interaction motifs in known APC/C substrates

Putative SUMO interaction motifs were predicted making use of the online tool GPS-SUMO [57] and setting a high threshold.

Substrate	UniProt ID	SUMO interaction motifs
Anillin [1]	Q9NQW6	DPKVEQKIEVIREIEMSVD (544 – 547)<br>DAANYYYLIILKAGAENMV (833 – 836)<br>ARRNTFELITVRPQREDDR (1058 – 1061)<br>MQKLNQVLVDIRLWQPDAC (1106 – 1109)
Aurora A [2]	O14965	None found
Aurora B [3]	Q96GD4	None found
B99 [4]	Q9NYZ3	DDPKKEDILLLADEKFDFD (9 – 12)<br>NRKTDSRLVDVSPDRGSPP (571 – 575)<br>VGSESRPLIDLMTNTPDMN (670 – 673)
BARD1 [5]	Q99728	GQRRDGPLVLIGSGLSSEQ (570 – 573)<br>DCVMSFELLPLDS****** (772 – 776)
Bub1 [6]	O43683	ESNMERRVITISKSEYSVH (211 – 215)<br>IRALRNRLIVLLLECKRSR (1073 – 1077)
Cdc6 [7]	Q99741	TAEKGPMIVLVLDEMDQLD (279 – 283)<br>LSNSHLVLIGIANTLDLTD (313 – 316)<br>LDVCRRAIEIVESDVKSQT (398 – 402)
Cdc20 [8]	Q12834	None found
Cdh1 [9]	Q9UM11	ERRLLRQIVIQNENTMPRV (13 – 15)<br>TKVKWESVSVLNLFTRIR* (486 – 489)
CDR2 [10]	Q01850	None found
CENPF [11]	P49454	None found
Centrin [12]	O15182	None found
Cdc25A [13]	P30304	VPTDGKRVIVVFHCEFSSE (425 – 428)
CDT1 [14]	Q9H211	None found
CKAP2 [15]	Q8WWK9	ITSPIENIIAIYEKAILAG (480 – 483)<br>IEEMRHTIVDILTMKSQEK (502 – 506)
CKS1 [16]	P61024	None found
Claspin [17]	Q9HAW4	None found
Cyclin A2 [18]	P20248	None found
Cyclin B1 [19]	P14635	PLEKVPMLVPVPVSEPVPE (89 – 92)<br>EKLSPEPILVDTASPSPME (120 – 124)
Cyclin B3 [20]	Q8WWL7	NIMEKPLILDISTTSKTPN (136 – 140)

		CDCEAQGLVL********* (1393 – 1395)
DRP1 [21]	O00429	DIIQLPQIVVVGTQSSGKS (28 – 31)<br>FPKLHDAIVEVVTCLLRKR (464 – 468)
E2C [22]	O00762	None found
E2F1 [23]	Q01094	RLLDSSQIVIISAAQDASA (33 – 37)<br>LSHSADGVVDLNWAAEVLK (150 – 153)
E2F3 [24]	O00716	SGLKDQTVIVVKAPPETRL (315 – 318)
ECT2 [25]	Q9H8V3	None found
FoxM1 [26]	Q08050	None found
G9A [27]	Q96KQ7	WAAEHKHIEVIRMLLTRGA (798 – 801)
Geminin [28]	O75496	None found
GLP1 [27]	Q9H9B1	None found
Glutaminase 1 [29]	O94925	None found
HEC1 [30]	O14777	None found
Hmmr [5]	O75330	KQSLEENIVILSKQVEDLN (279 – 283)
HSF2 [31]	Q03933	NMYGFRKVVHIDSGIVKQE (73 – 77)<br>RERISDDIIIYDVTDDNAD (246 – 250)<br>NCSQYPDIVIVEDDNEDEY (284 – 288)
HURP [5]	Q15398	PTLEGRILVELDETSQGLV (60 – 64)
ID2 [32]	Q02363	LQHVIDYILDLQIALDSHP (72 – 75)<br>ALDSHPTIVSLHHQRPGQN (85 – 88)
JNK1 [33]	P45983	None found
JNK2 [33]	P45984	None found
KID [34]	Q14807	ESFLKANILGLAAGQRCGA (653 – 656)
KIFC1 [35]	Q9BW19	None found
KIF18A [35]	Q8NI77	None found
KIF2C [35]	Q99661	RMHGKFSLVDLAGNERGAD (490 – 493)
KIF4A [35]	O95239	LEIYNEEILDLLCPSREKA (148 – 152)<br>DQELKENVEIICNLQQLIT (463 – 466)
MCL1 [36]	Q07820	DELYRQSLEIISRYLREQA (179 – 183)
MFN1 [37]	Q8IWA4	CALLRDDLVLVDSPGTDVT (174 – 178)<br>TSRTSMGIIIVGGVIWKTI (599 – 602)
MOAP-1 [38]	Q96BY2	None found
MPS1 [39]	P33981	None found
NEK2A [40]	P51955	None found
NIPA [41]	Q86WB0	None found
NLP [42]	Q9Y2I6	HDAQRKEIEVLKKDKEKAC (1121 – 1124)
NuSAP [5]	Q9BXS6	VLGMRRGLILAED****** (436 – 440)
OCT1 [43]	P14859	None found
OPA1 [37]	O60313	LIDMYSEVLDVLSDYDASY (271 – 275)

		TQDHLPRVVVVGDQSAGKT (291 - 294)
p21[44]	P38936	None found
p63 [45]	Q9H3D4	GMNRRPILIIVTLETRDGQ (322 - 326)
p190RhoGAP [46]	Q9NRY4	LAKTKKPIVVVLTKCDEGV (195 - 199)<br>SDPNIDRINLVILGKDGLA (598 - 602)<br>FLCEVQDIIPIQLVALTDG (880 - 883)<br>HDSTQGKIITIRNINKAQS (1116 - 1119)<br>ATRTYQTIIELFIQQCPFF (1424 - 1427)
PAF15 [47]	Q15004	None found
PFKFB3 [48]	Q16875	LTNSPTVIVMVGLPARGKT (38 - 41)<br>RDLSLIKVIDVGRRFLVNR (215 - 218)
PIF1 [49]	Q9H611	None found
PLK1 [50]	P53350	None found
RacGAP1 [51]	Q9H0H5	TSPMIPSIVVHCVNEIEQR (365 - 368)
RAP80 [52]	Q96RL1	None found
RASSF1A [53]	Q9NS23	None found
RCS1 [54]	Q9BSJ6	None found
Securin [55]	O95997	None found
SGO1 [56]	Q5FBB7	None found
SKP2 [16]	Q13309	None found
SnoN [57]	P12757	None found
Sororin [58]	Q96FF9	EAAEQFDLLVE******** (249 - 252)
Sp100 [59]	P23497	HHNQASDIIVISSEDSEGS (323 - 327)
TFAM [37]	Q00059	None found
TK1 [60]	P04183	None found
TMPK [61]	P23919	MAARRGALIVLEGVDRAGK (8 - 12)
TOME-1 [62]	Q99618	None found
TRB3 [63]	Q96RU7	EEEGDREVVLYG******* (354 - 357)
TPX2 [64]	Q9ULW0	None found
USP1 [65]	O94782	None found
USP37 [66]	Q86T82	EEELLAAVLEISKRDASPS (712 - 716)

Supplementary table 2: List of biotinylated peptides tested for binding to 3xSUMO2

protein	position	sequence	WT/mutant
KIF18B	aa 12-26	(Biotin)VEDSTLQVVVRVRPP	WT
KIF18B	aa 12-26	(Biotin)VEDSTAQAAARVRPP	mutant
KIF18B	aa 33-47	(Biotin)SQRRPVVQVVDERVL	WT
KIF18B	aa 33-47	(Biotin)SQRRPAAQAADERVL	mutant
KIF18B	aa 41-54	(Biotin)VVDERVLVFNPEEP	WT
KIF18B	aa 41-54	(Biotin)VVDERAAAFNPEEP	mutant
KIF18B	aa 167-181	(Biotin)VYNEQIHDLLEPKGP	WT
KIF18B	aa 167-181	(Biotin)VYNEQAHDAAEPKGP	mutant
KIF18B	aa 186-199	(Biotin)EDPDKGVVVQGLSF	WT
KIF18B	aa 186-199	(Biotin)EDPDKGAAAQGLSF	mutant
KIF18B	aa 203-217	(Biotin)ASAEQLLEILTRGNR	WT
KIF18B	aa 203-217	(Biotin)ASAEQAAEAATRGNR	mutant
KIF18B	aa 256-269	(Biotin)VAKMSLIDLAGSER	WT
KIF18B	aa 256-269	(Biotin)VAKMSAADAAGSER	mutant
KIF18B	aa 285-299	(Biotin)NINRSLLALINVLNA	WT
KIF18B	aa 285-299	(Biotin)NINRSAAAAANVLNA	mutant
KIF18B	aa 315-328	(Biotin)SKLTRLLKDSLGGN	WT
KIF18B	aa 315-328	(Biotin)SKLTRAAKDSLGGN	mutant
KIF18B	aa 355-369	(Biotin)DRAKEIRLSLKSNVT	WT
KIF18B	aa 355-369	(Biotin)DRAKEARASAKSNVT	mutant
KIF18B	aa 525-538	(Biotin)SKRLALKVLCVAQR	WT
KIF18B	aa 525-538	(Biotin)SKRLAAKAACVAQR	mutant
KIF18B	aa 821-836	(Biotin)RPAGPLVLPELPLSPL	WT
KIF18B	aa 821-836	(Biotin)RPAGPAAAPELPLSPL	mutant
RNF4	aa 41-54	(Biotin)TAGDEIVDLTCESL	WT
RNF4	aa 41-54	(Biotin)TAGDEAADATCESL	mutant
RNF4	aa 53-66	(Biotin)SLEPVVVDLTHNDS	WT
RNF4	aa 53-66	(Biotin)SLEPVAADATHNDS	mutant

Supplementary References

1. Zhao WM, Fang G. Anillin is a substrate of anaphase-promoting complex/cyclosome (APC/C) that controls spatial contractility of myosin during late cytokinesis. *The Journal of biological chemistry* **280**, 33516-33524 (2005).

2. Littlepage LE, Ruderman JV. Identification of a new APC/C recognition domain, the A box, which is required for the Cdh1-dependent destruction of the kinase Aurora-A during mitotic exit. *Genes & development* **16**, 2274-2285 (2002).

3. Stewart S, Fang G. Destruction box-dependent degradation of aurora B is mediated by the anaphase-promoting complex/cyclosome and Cdh1. *Cancer research* **65**, 8730-8735 (2005).

4. Pfleger CM, Kirschner MW. The KEN box: an APC recognition signal distinct from the D box targeted by Cdh1. *Genes & development* **14**, 655-665 (2000).

5. Song L, Rape M. Regulated degradation of spindle assembly factors by the anaphase-promoting complex. *Molecular cell* **38**, 369-382 (2010).

6. Qi W, Yu H. KEN-box-dependent degradation of the Bub1 spindle checkpoint kinase by the anaphase-promoting complex/cyclosome. *The Journal of biological chemistry* **282**, 3672-3679 (2007).

7. Petersen BO*, et al.* Cell cycle- and cell growth-regulated proteolysis of mammalian CDC6 is dependent on APC-CDH1. *Genes & development* **14**, 2330-2343 (2000).

8. Ge S, Skaar JR, Pagano M. APC/C- and Mad2-mediated degradation of Cdc20 during spindle checkpoint activation. *Cell cycle* **8**, 167-171 (2009).

9. Listovsky T*, et al.* Mammalian Cdh1/Fzr mediates its own degradation. *The EMBO journal* **23**, 1619-1626 (2004).

10. O'Donovan KJ, Diedler J, Couture GC, Fak JJ, Darnell RB. The onconeural antigen cdr2 is a novel APC/C target that acts in mitosis to regulate c-myc target genes in mammalian tumor cells. *PloS one* **5**, e10045 (2010).

11. Gurden MD*, et al.* Cdc20 is required for the post-anaphase, KEN-dependent degradation of centromere protein F. *Journal of cell science* **123**, 321-330 (2010).

12. Lukasiewicz KB, Greenwood TM, Negron VC, Bruzek AK, Salisbury JL, Lingle WL. Control of centrin stability by Aurora A. *PloS one* **6**, e21291 (2011).

13. Donzelli M, Squatrito M, Ganoth D, Hershko A, Pagano M, Draetta GF. Dual mode of degradation of Cdc25 A phosphatase. *The EMBO journal* **21**, 4875-4884 (2002).

14. Sugimoto N*, et al.* Identification of novel human Cdt1-binding proteins by a proteomics approach: proteolytic regulation by APC/CCdh1. *Molecular biology of the cell* **19**, 1007-1021 (2008).

15. Seki A, Fang G. CKAP2 is a spindle-associated protein degraded by APC/C-Cdh1 during mitotic exit. *The Journal of biological chemistry* **282**, 15103-15113 (2007).

16. Bashir T, Dorrello NV, Amador V, Guardavaccaro D, Pagano M. Control of the SCF(Skp2-Cks1) ubiquitin ligase by the APC/C(Cdh1) ubiquitin ligase. *Nature* **428**, 190-193 (2004).

17. Faustrup H, Bekker-Jensen S, Bartek J, Lukas J, Mailand N. USP7 counteracts SCFbetaTrCP- but not APCCdh1-mediated proteolysis of Claspin. *The Journal of cell biology* **184**, 13-19 (2009).

18. Geley S, Kramer E, Gieffers C, Gannon J, Peters JM, Hunt T. Anaphase-promoting complex/cyclosome-dependent proteolysis of human cyclin A starts at the beginning of mitosis and is not subject to the spindle assembly checkpoint. *The Journal of cell biology* **153**, 137-148 (2001).

19. Clute P, Pines J. Temporal and spatial control of cyclin B1 destruction in metaphase. *Nature cell biology* **1**, 82-87 (1999).

20. Nguyen TB*, et al.* Characterization and expression of mammalian cyclin b3, a prepachytene meiotic cyclin. *The Journal of biological chemistry* **277**, 41960-41969 (2002).

21. Horn SR*, et al.* Regulation of mitochondrial morphology by

APC/CCdh1-mediated control of Drp1 stability. *Molecular biology of the cell* **22**, 1207-1216 (2011).

22. Rape M, Kirschner MW. Autonomous regulation of the anaphase-promoting complex couples mitosis to S-phase entry. *Nature* **432**, 588-595 (2004).

23. Peart MJ*, et al.* APC/C(Cdc20) targets E2F1 for degradation in prometaphase. *Cell cycle* **9**, 3956-3964 (2010).

24. Ping Z, Lim R, Bashir T, Pagano M, Guardavaccaro D. APC/C (Cdh1) controls the proteasome-mediated degradation of E2F3 during cell cycle exit. *Cell cycle* **11**, 1999-2005 (2012).

25. Liot C, Seguin L, Siret A, Crouin C, Schmidt S, Bertoglio J. APC(cdh1) mediates degradation of the oncogenic Rho-GEF Ect2 after mitosis. *PloS one* **6**, e23676 (2011).

26. Laoukili J, Alvarez-Fernandez M, Stahl M, Medema RH. FoxM1 is degraded at mitotic exit in a Cdh1-dependent manner. *Cell cycle* **7**, 2720-2726 (2008).

27. Takahashi A*, et al.* DNA damage signaling triggers degradation of histone methyltransferases through APC/C(Cdh1) in senescent cells. *Molecular cell* **45**, 123-131 (2012).

28. McGarry TJ, Kirschner MW. Geminin, an inhibitor of DNA replication, is degraded during mitosis. *Cell* **93**, 1043-1053 (1998).

29. Colombo SL*, et al.* Anaphase-promoting complex/cyclosome-Cdh1 coordinates glycolysis and glutaminolysis with transition to S phase in human T lymphocytes. *Proceedings of the National Academy of Sciences of the United States of America* **107**, 18868-18873 (2010).

30. Li L*, et al.* Anaphase-promoting complex/cyclosome controls HEC1 stability. *Cell proliferation* **44**, 1-9 (2011).

31. Ahlskog JK*, et al.* Anaphase-promoting complex/cyclosome participates in the acute response to protein-damaging stress. *Molecular and cellular biology* **30**, 5608-5620 (2010).

32. Lasorella A*, et al.* Degradation of Id2 by the anaphase-promoting complex couples cell cycle exit and axonal growth. *Nature* **442**, 471-474 (2006).

33. Gutierrez GJ, Tsuji T, Chen M, Jiang W, Ronai ZA. Interplay between Cdh1 and JNK activity during the cell cycle. *Nature cell biology* **12**, 686-695 (2010).

34. Feine O, Zur A, Mahbubani H, Brandeis M. Human Kid is degraded by the APC/C(Cdh1) but not by the APC/C(Cdc20). *Cell cycle* **6**, 2516-2523 (2007).

35. Singh SA*, et al.* Co-regulation proteomics reveals substrates and mechanisms of APC/C-dependent degradation. *The EMBO journal* **33**, 385-399 (2014).

36. Harley ME, Allan LA, Sanderson HS, Clarke PR. Phosphorylation of Mcl-1 by CDK1-cyclin B1 initiates its Cdc20-dependent destruction during mitotic arrest. *The EMBO journal* **29**, 2407-2420 (2010).

37. Garedew A, Andreassi C, Moncada S. Mitochondrial dynamics, biogenesis, and function are coordinated with the cell cycle by APC/C CDH1. *Cell metabolism* **15**, 466-479 (2012).

38. Huang NJ, Zhang L, Tang W, Chen C, Yang CS, Kornbluth S. The Trim39 ubiquitin ligase inhibits APC/CCdh1-mediated degradation of the Bax activator MOAP-1. *The Journal of cell biology* **197**, 361-367 (2012).

39. Cui Y*, et al.* Degradation of the human mitotic checkpoint kinase Mps1 is cell cycle-regulated by APC-cCdc20 and APC-cCdh1 ubiquitin ligases. *The Journal of biological chemistry* **285**, 32988-32998 (2010).

40. Hegemann B*, et al.* Systematic phosphorylation analysis of human mitotic protein complexes. *Science signaling* **4**, rs12 (2011).

41. Klitzing C*, et al.* APC/C(Cdh1)-mediated degradation of the F-box protein NIPA is regulated by its association with Skp1. *PloS one* **6**, e28998 (2011).

42. Wang Y, Zhan Q. Cell cycle-dependent expression of centrosomal ninein-like protein in human cells is regulated by the anaphase-promoting complex. *The Journal of biological chemistry* **282**, 17712-17719 (2007).

43. Kang J, Goodman B, Zheng Y, Tantin D. Dynamic regulation of Oct1 during mitosis by phosphorylation and ubiquitination. *PloS one* **6**, e23872 (2011).

44. Amador V, Ge S, Santamaria PG, Guardavaccaro D, Pagano M. APC/C(Cdc20) controls the ubiquitin-mediated degradation of p21 in prometaphase. *Molecular cell* **27**, 462-473 (2007).

45. Hau PM, Yip YL, Huen MS, Tsao SW. Loss of DeltaNp63alpha promotes mitotic exit in epithelial cells. *FEBS letters* **585**, 2720-2726 (2011).

46. Naoe H*, et al.* The anaphase-promoting complex/cyclosome activator Cdh1 modulates Rho GTPase by targeting p190 RhoGAP for degradation. *Molecular and cellular biology* **30**, 3994-4005 (2010).

47. Emanuele MJ, Ciccia A, Elia AE, Elledge SJ. Proliferating cell nuclear antigen (PCNA)-associated KIAA0101/PAF15 protein is a cell cycle-regulated anaphase-promoting complex/cyclosome substrate. *Proceedings of the National Academy of Sciences of the United States of America* **108**, 9845-9850 (2011).

48. Herrero-Mendez A, Almeida A, Fernandez E, Maestre C, Moncada S, Bolanos JP. The bioenergetic and antioxidant status of neurons is controlled by continuous degradation of a key glycolytic enzyme by APC/C-Cdh1. *Nature cell biology* **11**, 747-752 (2009).

49. Mateyak MK, Zakian VA. Human PIF helicase is cell cycle regulated and associates with telomerase. *Cell cycle* **5**, 2796-2804 (2006).

50. Lindon C, Pines J. Ordered proteolysis in anaphase inactivates Plk1 to contribute to proper mitotic exit in human cells. *The Journal of cell biology* **164**, 233-241 (2004).

51. Min M, Mayor U, Dittmar G, Lindon C. Using in vivo biotinylated ubiquitin to describe a mitotic exit ubiquitome from human cells. *Molecular & cellular proteomics : MCP* **13**, 2411-2425 (2014).

52. Cho HJ*, et al.* Degradation of human RAP80 is cell cycle regulated by Cdc20 and Cdh1 ubiquitin ligases. *Molecular cancer research : MCR* **10**, 615-625 (2012).

53. Chow C*, et al.* Regulation of APC/CCdc20 activity by

RASSF1A-APC/CCdc20 circuitry. *Oncogene* **31**, 1975-1987 (2012).

54. Zhao WM, Coppinger JA, Seki A, Cheng XL, Yates JR, 3rd, Fang G. RCS1, a substrate of APC/C, controls the metaphase to anaphase transition. *Proceedings of the National Academy of Sciences of the United States of America* **105**, 13415-13420 (2008).

55. Hagting A, Den Elzen N, Vodermaier HC, Waizenegger IC, Peters JM, Pines J. Human securin proteolysis is controlled by the spindle checkpoint and reveals when the APC/C switches from activation by Cdc20 to Cdh1. *The Journal of cell biology* **157**, 1125-1137 (2002).

56. Karamysheva Z, Diaz-Martinez LA, Crow SE, Li B, Yu H. Multiple anaphase-promoting complex/cyclosome degrons mediate the degradation of human Sgo1. *The Journal of biological chemistry* **284**, 1772-1780 (2009).

57. Stroschein SL, Bonni S, Wrana JL, Luo K. Smad3 recruits the anaphase-promoting complex for ubiquitination and degradation of SnoN. *Genes & development* **15**, 2822-2836 (2001).

58. Rankin S, Ayad NG, Kirschner MW. Sororin, a substrate of the anaphase-promoting complex, is required for sister chromatid cohesion in vertebrates. *Molecular cell* **18**, 185-200 (2005).

59. Wang R, Li KM, Zhou CH, Xue JL, Ji CN, Chen JZ. Cdc20 mediates D-box-dependent degradation of Sp100. *Biochemical and biophysical research communications* **415**, 702-706 (2011).

60. Ke PY, Chang ZF. Mitotic degradation of human thymidine kinase 1 is dependent on the anaphase-promoting complex/cyclosome-CDH1-mediated pathway. *Molecular and cellular biology* **24**, 514-526 (2004).

61. Ke PY, Kuo YY, Hu CM, Chang ZF. Control of dTTP pool size by anaphase promoting complex/cyclosome is essential for the maintenance of genetic stability. *Genes & development* **19**, 1920-1933 (2005).

62. Ayad NG, Rankin S, Murakami M, Jebanathirajah J, Gygi S, Kirschner MW. Tome-1, a trigger of mitotic entry, is degraded during G1 via the APC. *Cell* **113**, 101-113 (2003).

63. Ohoka N, Sakai S, Onozaki K, Nakanishi M, Hayashi H. Anaphase-promoting complex/cyclosome-cdh1 mediates the ubiquitination and degradation of TRB3. *Biochemical and biophysical research communications* **392**, 289-294 (2010).

64. Stewart S, Fang G. Anaphase-promoting complex/cyclosome controls the stability of TPX2 during mitotic exit. *Molecular and cellular biology* **25**, 10516-10527 (2005).

65. Cotto-Rios XM, Jones MJ, Busino L, Pagano M, Huang TT. APC/CCdh1-dependent proteolysis of USP1 regulates the response to UV-mediated DNA damage. *The Journal of cell biology* **194**, 177-186 (2011).

66. Huang X*, et al.* Deubiquitinase USP37 is activated by CDK2 to antagonize APC(CDH1) and promote S phase entry. *Molecular cell* **42**, 511-523 (2011).
